# The End of Silo Medicine: A Case for Disease‐Centered Subspecialization

**DOI:** 10.1111/jep.70542

**Published:** 2026-07-27

**Authors:** Thomas Hegyi, Robert J. Ostfeld

**Affiliations:** ^1^ Department of Pediatrics, Robert Wood Johnson Medical School, Division of Neonatology Rutgers University New Brunswick New Jersey USA; ^2^ Department of Medicine, Division of Cardiology Montefiore Health System Bronx New York USA

## Abstract

Modern medical subspecialization was organized around organs, age groups and procedures during an era when diseases were viewed as isolated processes. Contemporary biology instead reveals illnesses that arise across systems and evolve across the life span. Yet clinical care remains fragmented among multiple specialties with no single physician accountable for the integrated whole. We propose disease‐centred subspecialization, in which expertise for a complex condition is unified within a single clinical domain and supported by advanced clinical intelligence systems. Fetoneonatology, integrating foetal and neonatal medicine across the continuum of pregnancy and early life, illustrates this model. Similar integration is urgently needed for metabolic syndrome and ischaemic heart disease, conditions whose pathophysiology spans multiple organs and specialties. Evidence increasingly shows that fragmented care contributes to medical error, contradictory treatment plans, rehospitalization and poor long‐term outcomes. We outline a framework for training, certification and AI augmented practice, organized around diseases rather than anatomy alone.

1

The organization of modern medicine emerged from the scientific reforms initiated by the Flexner Report, which standardized medical education and fostered organ‐based specialization [1]. Cardiology focuses on the heart, nephrology on the kidney and pulmonology on the lung. This structure was effective when diseases were viewed largely as isolated organ disorders and therapeutic options were limited.

Contemporary disease biology no longer conforms to these boundaries. Patients increasingly present with interconnected conditions involving multiple organs, overlapping mechanisms and longitudinal trajectories spanning decades. A patient with type 2 diabetes, obesity, hypertension, steatohepatitis, sleep apnoea and ischaemic heart disease may simultaneously require endocrinology, cardiology, hepatology, nephrology, sleep medicine and primary care. No single physician is formally trained or accountable for integrating the totality of the disease process. The result is duplication, omission, contradictory treatment plans and preventable harm.

Bodenheimer described the American patient's experience as a ‘perilous journey through the health care system’, emphasizing that failures in coordination are structural rather than individual [2]. Stange similarly identified fragmentation as a defining pathology of modern medicine [3]. We propose complementing existing specialties with disease‐centred subspecialties that assume integrative responsibility for complex multisystem disorders.

Fetoneonatology illustrates this concept. Neonatal diseases often originate before birth through placental dysfunction, inflammatory signalling, maternal illness, epigenetic programming and altered foetal growth. Bronchopulmonary dysplasia, necrotizing enterocolitis and intraventricular haemorrhage are not exclusively neonatal disorders but foetal‐neonatal conditions whose biology spans pregnancy and postnatal life.

Despite this continuity, medicine imposes a sharp professional division at the point of delivery. Maternal–foetal medicine specialists oversee prenatal care while neonatologists assume responsibility after birth. Neither discipline requires mastery of the other's domain, and the placenta belongs fully to neither. Yet landmark advances emerged precisely from crossing this boundary. Liggins and Howie demonstrated that antenatal corticosteroids reduce the incidence of neonatal respiratory distress syndrome, thereby linking obstetrics directly to neonatal outcomes [4]. Barker later showed that intrauterine conditions influence adult cardiovascular and metabolic disease, establishing the developmental origins of health and disease [5, 6].

We had proposed fetoneonatology as a formal subspecialty integrating placentation, foetal physiology, foetal surveillance, preterm birth biology, neonatal adaptation and the long‐term consequences of prenatal exposures. Such specialists would synthesize maternal, placental, foetal, genomic and neonatal data into a unified clinical framework, increasingly supported by advanced AI systems capable of longitudinal analysis.

The same need for integration exists in metabolic syndrome. More than one‐third of American adults meet diagnostic criteria for this condition, which underlies cardiovascular disease, diabetes, steatohepatitis, chronic kidney disease and stroke [7]. Its mechanisms, including inflammation, insulin resistance, adipokine dysregulation and mitochondrial dysfunction, operate simultaneously across organ systems.

Yet management remains fragmented. Endocrinologists manage glycemic control, cardiologists address hypertension and dyslipidemia, hepatologists treat steatohepatitis, nephrologists intervene when renal disease develops and primary care physicians attempt coordination. Boyd and colleagues demonstrated that adherence to multiple disease‐specific guidelines frequently produces contradictory regimens for patients with multimorbidity [8]. Jencks and colleagues further showed that nearly one‐fifth of Medicare patients are rehospitalized within 30 days, with inadequate coordination playing a major role [9]. Although newer cardiovascular, kidney and metabolic initiatives acknowledge multisystem disease, current training pathways remain largely siloed [10, 11, 12, 13].

A disease‐centred metabolic specialist would integrate adiposity biology, vascular disease, insulin signalling, renal dysfunction, hepatic disease and preventive therapeutics within a single accountable framework. Such a role would not replace consultants but would unify fragmented management.

Ischaemic heart disease provides another example. Although interventional cardiology has transformed acute survival, chronic management often remains divided among imaging specialists, lipidologists, endocrinologists, rehabilitation experts, nephrologists, behavioural medicine physicians and preventive cardiologists [14, 15, 16, 17]. Yet plaque instability, inflammation, genetics, metabolic dysfunction and lifestyle behaviours are deeply interconnected determinants of outcome.

A disease‐centred specialist in ischaemic heart disease would integrate molecular mechanisms of atherosclerosis, preventive therapy, behavioural adherence, metabolic disease, imaging interpretation and rehabilitation science. AI‐supported systems could synthesize biomarker trajectories, imaging findings, genomic profiles and pharmacologic responses in real time, advancing what Topol termed ‘high‐performance medicine’ [18].

We do not propose a separate specialty for every disorder. Rather, disease‐centred subspecialization should be considered when optimal management requires knowledge distributed across several specialties, with no single physician accountable for integration.

Implementing this model would require substantial changes in training and certification. Physicians could complete traditional residencies followed by cross‐disciplinary fellowships organized around diseases rather than organs. Certification should emphasize integrative clinical reasoning and management of multisystem complexity rather than isolated organ expertise. Training in AI‐assisted clinical decision‐making, including recognition of algorithmic bias and data limitations, should become essential.

Critics may argue that disease‐centred specialization sacrifices depth for breadth. We would argue the opposite. Depth remains essential, but its focus shifts from anatomy to disease biology. A fetoneonatologist would possess deeper integrated expertise in placental biology, foetal physiology and neonatal disease than current pathways permit.

Others may fear that new specialties would worsen fragmentation. In reality, the current problem derives from anatomical boundaries that divide inherently unified diseases. Disease‐centred specialists would reduce fragmentation by decreasing the number of disconnected consultants required for a single patient.

Finally, critics may note the absence of randomized trials comparing integrated and fragmented specialty models. Such evidence should indeed be generated through carefully designed pilot programmes. However, the lack of trial data does not validate the current structure, particularly when evidence already demonstrates the harms of poor coordination and multimorbidity‐driven fragmentation.

Medicine has repeatedly reorganized itself in response to scientific advances. Germ theory reshaped surgery, and molecular biology transformed oncology. Modern understanding of multisystem disease now requires a comparable rethinking of specialty practice. Patients do not experience disease in organ‐based silos. The organization of medicine should no longer do so either.

## Funding

The authors have nothing to report.

## Conflicts of Interest

The authors declare no conflicts of interest.

## Data Availability

The data that support the findings of this study are available on request from the corresponding author. The data are not publicly available due to privacy or ethical restrictions.
